# In-office Eustachian tube balloon dilation under local anesthesia as a response to operating room restrictions associated with the COVID-19 pandemic

**DOI:** 10.3389/fsurg.2023.1033010

**Published:** 2023-04-11

**Authors:** Sean C. Sheppard, Sven Beckmann, Marco Caversaccio, Lukas Anschuetz

**Affiliations:** Department of Otorhinolaryngology, Head and Neck Surgery, Inselspital, Bern University Hospital, Bern, Switzerland

**Keywords:** Eustachian tube, balloon dilation, Eustachian tube dysfunction, in-office, COVID-19, local anesthesia, ETDQ-7, operating room restrictions

## Abstract

**Objective:**

To evaluate the feasibility of local anesthesia for Eustachian tube balloon dilation as an in-office procedure for the treatment of Eustachian tube dilatory dysfunction as a response to the restriction measures of the coronavirus disease 2019 pandemic.

**Method:**

Patients with Eustachian tube dilatory dysfunction refractory to nasal steroids undergoing Eustachian tube balloon dilation in local anesthesia were enrolled in a prospective observational cohort between May 2020 and April 2022. The patients were assessed by using the Eustachian tube dysfunction questionnaire (ETDQ-7) score and Eustachian tube mucosal inflammation scale. They underwent clinical examination, tympanometry, and pure tone audiometry. Eustachian tube balloon dilation was performed in-office under local anesthesia. The perioperative experience of the patients was recorded using a 1–10 visual analog scale (VAS).

**Results:**

Thirty patients (47 Eustachian tubes) underwent the operation successfully. One attempted dilation was aborded because the patient displayed anxiety. Local anesthesia was performed by using topical lidocaine and nasal packing for all patients. Three patients required an infiltration of the nasal septum and/or tubal nasopharyngeal orifice. The mean time of the operation was 5.7 min per Eustachian tube dilation. The mean level of discomfort during the intervention was 4.7 (on a 1–10 VAS scale). All patients returned home immediately after the intervention. The only reported complication was a self-limiting subcutaneous emphysema.

**Conclusion:**

Eustachian tube balloon dilation can be performed under local anesthesia and is well tolerated by most patients. In the patients reported in this study, no major complications occurred. In order to free operation room capacities, the intervention can be performed in an in-office setting with satisfactory patient feedback.

## Introduction

Eustachian tube dysfunction (ETD) is a ubiquitous healthcare problem with a prevalence rate of 0.9% (1). The Eustachian tube is a partly fibrocartilaginous, partly bony canal connecting the middle ear to the nasopharynx. It has three main functions: equalization of the middle ear pressure, mucus drainage, and protection of the middle ear from nasopharyngeal pathogens, secretions, and sounds. The middle ear pressure adjusts with the atmosphere pressure during ventilation with Valsalva maneuvers, yawning, or swallowing. The mucosa of the tube consists of ciliated cells from the tympanic cavity to the pharynx that drain mucus from the middle ear (2).

Eustachian tube ventilation disorders can be categorized into obstructive and dynamic disorders, with a further classification of functional obstructive disorders based on the Eustachian tube mucosal inflammation scale (ETMIS) (3). Acute obstructive ETD (OETD) can develop secondary to upper respiratory tract infections and is usually resolved spontaneously. However, chronic forms may, in some cases, last for months to years, potentially resulting in severe consequences for the middle ear function, such as hearing loss, chronic effusion, or cholesteatoma. The other causes of OETD are sinonasal diseases, gastroesophageal reflux, or a nasopharyngeal mass. The common symptoms of OETD are aural fullness, aural pressure, hearing loss, and otalgia, which necessitate an exclusion of patulous ETD, temporomandibular joint disorders, extrinsic obstruction, superior semicircular canal dehiscence, and endolymphatic hydrops (4). The severity and course of symptoms can be assessed using validated questionnaires such as the Eustachian tube dysfunction questionnaire (ETDQ-7) (5). As a first-line treatment of chronic OETD, topical nasal steroids are habitually proposed, although concrete evidence of their therapeutic effects is not well-established (6). In cases where the symptoms persist, surgical therapy such as balloon dilatation of the Eustachian tube (BDET) has been shown as a valid and effective alternative. Nowadays, there is increasing evidence on the effectiveness of balloon dilatation of the Eustachian tube (7). However, to this date, the procedure is mainly performed under general anesthesia (8), although its application in local anesthesia has also been reported (9, 10).

The spread of the coronavirus disease 2019 (COVID-19) resulted in significant changes in the management of otorhinolaryngologic cases. In particular, functional surgeries including BDET were often canceled or postponed to allow for sufficient healthcare resources to be attributed to infected patients, as well as emergency and oncological surgeries (11). Despite the closure of operating theaters and reduction of healthcare resources, in-office procedures remained available. As a result of these conditions, we altered our BDET procedure, moving away from general anesthesia to awake and local anesthesia and in-office BDET. In this article, we describe our preliminary results of in-office BDET and discuss the advantages and drawbacks of this approach followed during the COVID-19 pandemic.

## Patients and methods

### Clinical data

The local ethics committee approved this study (KEK 2019-00555). Thirty patients (47 Eustachian tubes) were enrolled in a prospective observational cohort study between May 2020 and April 2022; they were undergoing in-office Eustachian tube balloon dilatation in our tertiary referral center. Patients suffering from Eustachian tube dysfunction and who were refractory to topical nasal steroid therapy for at least 3 months were included. Diagnosis was based on the presence of symptoms such as pressure imbalances, aural fullness, popping, and discomfort/pain even with normal ETDQ scores or normal findings on clinical examination or audiometric tests. Numerous patients reported symptoms only when they experienced altitude changes, and thus, these were consistent with baro-challenge-induced eustachian tube dysfunction. Patients suspected of patulous Eustachian tube dysfunction with symptoms of autophony or mobile tympanic membrane during forced respiration were excluded. One procedure was aborted because of a high anxiety level of the patient and a very narrow nasal anatomy and was thus not included in the analysis.

Preoperative assessment of patients consisted of a record of complete history, ETDQ-7 score, ETMIS, and a complete clinical examination. Subjective and objective Valsalva maneuvers were evaluated, as well as transnasal nasopharyngoscopy with 30° endoscopes. In addition, tympanometry and pure tone audiometry were performed. Particular attention was directed toward possible obstructions in the transnasal approach, such as septal deviations or spurs as well as inferior turbinate hypertrophy. Discomfort during surgery was measured using the VAS. A follow-up examination was performed 3 months later using transnasal nasopharyngoscopy, ETDQ-7 score, and ETMIS. Using the VAS, each patient was asked whether they had any symptoms of discomfort preoperatively and at follow-up. The patients were also asked about their levels of satisfaction with the intervention at follow-up on a VAS.

### Statistical analysis

Clinical characteristics and operation reports were recorded and collected in a study database. Statistical analysis was performed using the SPSS statistics software Version 25 (IBM, Armonk, NY, United States) and plotted with GraphPad Prism 9 (GraphPad Software, Inc., San Diego, CA, United States). Comparisons were performed by using the paired *T*-test for continuous variables and the McNemar test for categorical variables. A statistical analysis of audiometric data was performed when both pre- and postoperative data were available. All tests were two-sided, and statistical significance was determined by using a *P*-value of <0.05.

### Surgical protocol

Due to restricted capacity in the operating theater, the surgery was performed as an in-office procedure. The patients were not premedicated before the intervention and a 30-min time slot was fixed for them. Preoperative COVID-19 antigen or PCR testing was not required according to local hospital policy and only asymptomatic patients were allowed for surgery. The patients were positioned in a semisitting position with a head elevation of approximately 15°. The COVID-19 pandemic warranted the face mask to be kept over the mouth at all times. Moreover, appropriate personal protective equipment was worn by the performing physicians.

#### Local anesthesia

Two puffs of xylocain spray 10% were sprayed into the indicated nostril. Afterward, nasal packing soaked in oxybuprocain 1% and xylometazoline 0.1% for a duration of 15 min in the indicated nostril was applied. For patients with a narrow nose, local anesthesia was applied in the contralateral nostril for keeping open the possibility of passing an angled endoscope through the contralateral nostril. Removal of the nasal packing allowed the start of the surgical procedure. If required, infiltration anesthesia was performed by administering 1% rapidocaine.

#### Intervention

**Figure 1:**
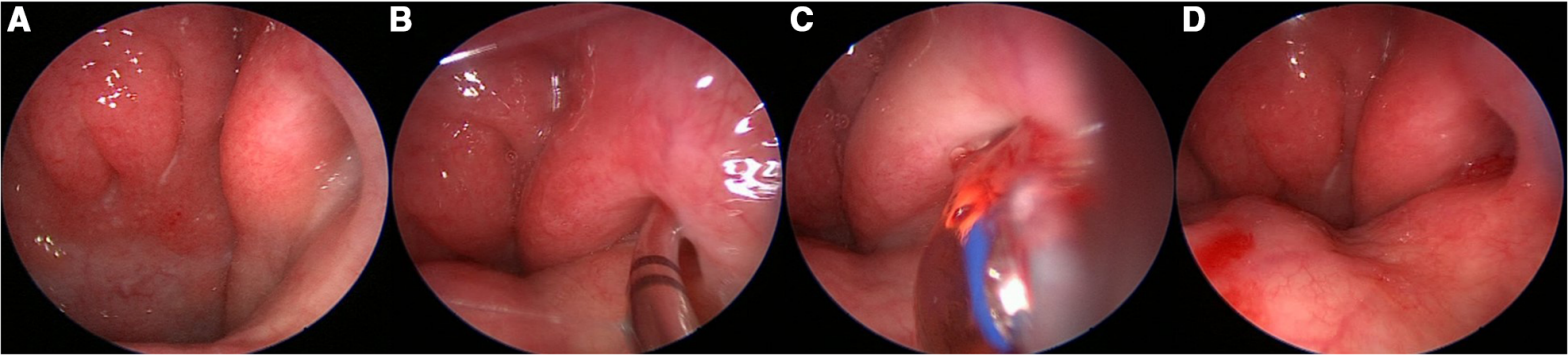
(**A**) Endoscopy of a grade II Eustachian tube. (**B**) Entering and advancing the cartilaginous part of the Eustachian tube. (**C**) Blowing the balloon. (**D**) End of the operation; no bleeding is noticed.

First, transnasal nasopharyngoscopy was performed and, if appropriate, septal spurs were infiltrated. Insertion of the Eustachian tube dilatory device, XprESS ENT Dilation system (Stryker, Kalamazoo, MI, United States), was performed with the instrument tip directed downward along the nasal floor into the nasopharynx under endoscopic control with a 0°, 3-mm-diameter, and 14-cm-long optic coupled to a high-definition video system and screen (Karl Storz, Tuttlingen, Germany). The 0° endoscopes were preferably used for a large field of view of the endonasal and nasopharyngeal structures. Scopes with 30° and 45° angles were also available if needed. The dilatation device consisting of the guide and balloon was placed under the endoscope and carefully introduced to not injure the mucosa. After a complete introduction of the device in the nasopharynx, a rotational movement was performed to introduce the 45° angled guide into the eustachian tube orifice under endoscopic view. Only light pressure was applied, and a tactile feedback of the tip of the device helped the surgeon to ensure the right position. Finally, the balloon was advanced into the eustachian tube orifice. The balloon was inflated at 10 bars for 2 min, while the Eustachian tube orifice was endoscopically observed. Afterward, the balloon was deflated and retracted under endoscopic control after rotary movement in the nasopharynx along the nasal floor. To finalize the procedure, the Eustachian tube was endoscopically examined for the presence of blood or fluid secretions (Figure 1).

## Results

In total, 30 operations including 47 Eustachian tubes were successfully performed under local anesthesia. We report one failure of BDET due to patient anxiety during the performance of nasal endoscopy, and this case was excluded from the analysis. The intervention was canceled and rescheduled under general anesthesia. The patients’ clinical and operating features are reported in Table 1. The mean age was 40 years (min. 18; max. 79 years) with 13 female participants (43%). Local anesthesia with topical lidocaine and nasal packing was administered in all patients. Three patients (10%) required additional infiltration anesthesia of the nasal septum and of the Eustachian tube.

**Table 1:** Patients’ clinical and operative features.

Features	*N* = 30	%
Age mean (min., max.)	40.3 (18–79)	
Gender		
Male	17	57
Female	13	43
Previous otologic disease		
Yes	21	70
No	9	30
Anesthesia type		
Spray + nasal packing	30	100
Spray + nasal packing + infiltration	3	10
Complications		
Emphysema	1	3
Satisfied, would recommend to a friend		
Yes	30	100

The mean time of intervention was 5.7 min with a mean discomfort of 4.7 recorded on the VAS during the intervention (1–10). In the self-questionnaire, all 30 patients answered that they were satisfied and would recommend the procedure under local anesthesia to a friend. All patients were able to be discharged home immediately after the procedure. No major complications were observed; however, we noted the occurrence of one minor complication in the form of a self-limiting subcutaneous cervico-facial emphysema.

**Figure 2:**
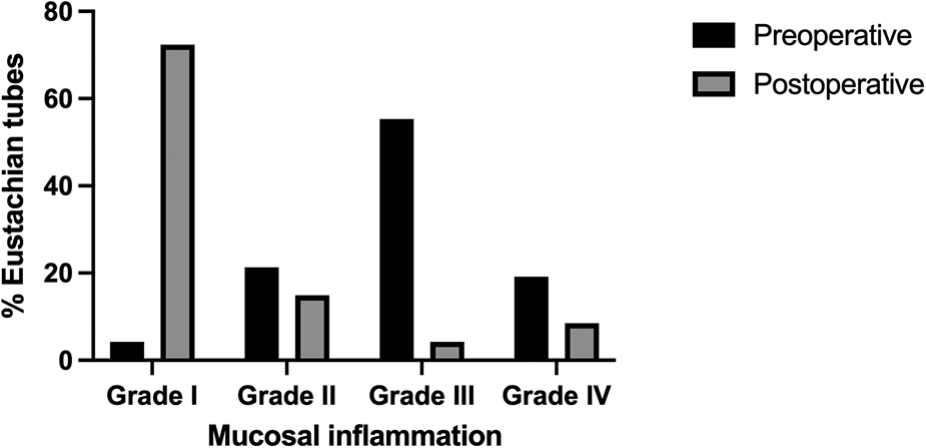
Eustachian tube mucosal inflammation scale preoperatively and at a 3-month follow-up.

**Figure 3:**
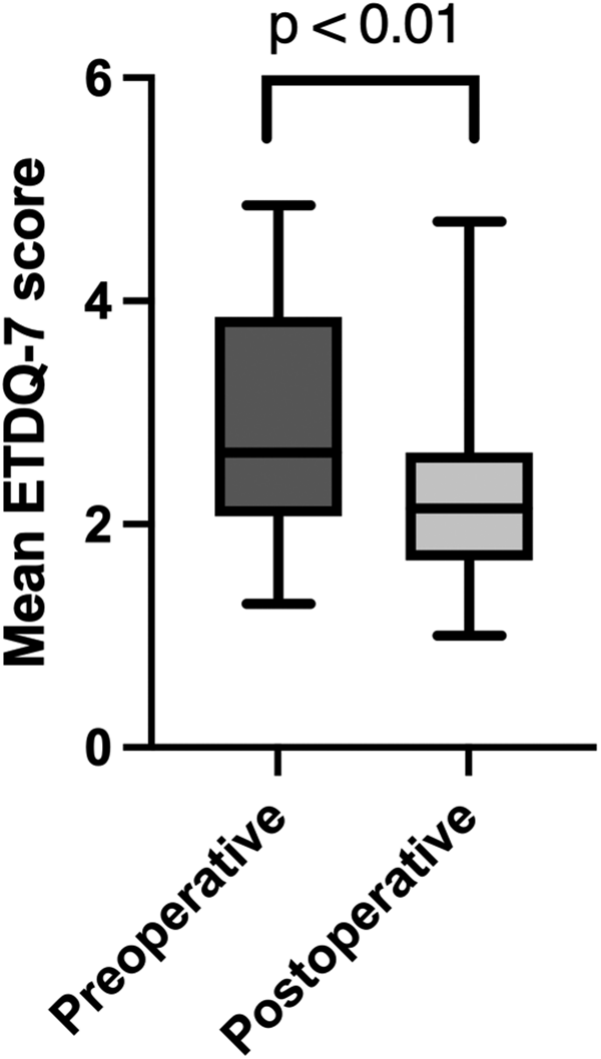
Mean overall ETDQ-7 score preoperatively and at a 3-month follow-up.

The median follow-up time, from the operation date to the first follow-up consultation, was 15.4 weeks. Individual patients with ETDQ-7 and ETMIS grades are reported in Table 2. Preoperatively, 26 Eustachian tubes (55.3%) presented with ETMIS grade III, followed by 10 (21.3%) with ETMIS grade II and 9 (19.2%) with ETMIS grade IV. In the postoperative follow-up 3 months later, most patients (72.3%) showed ETMIS grade I, followed by 7 who showed ETMIS grade II (14.9%) (*P *< 0.001) (Figure 2). The objective Valsalva maneuver was improved from 24 to 35 ET at the follow-up (*P *< 0.01). The 3-month mean overall ETDQ-7 score was reduced from 2.83 ± 1.052 to 2.14 ± 0.85 (*P *< 0.01), as shown in Figure 3. On a VSA (0–10), patients reported preoperative discomfort at a median of 6 (IQR = 4) and postoperative discomfort at 3 (IQR = 4.5). The level of satisfaction with the intervention at 3 months was reported at a median of 7 (IQR = 3).

**Table 2:** Individual patients’ mean ETDQ-7 score and ETMIS grade preoperatively and at a 3-month follow-up.

Patient	Preoperative				Follow-up at 3 months			
	Mean ETDQ-7 score	ETMIS grade	PTA (ACT)	Tymp	Mean ETDQ-7 score	ETMIS grade	PTA (ACT)	Tymp
1	1.43	R = 3; L = 3	R = NA; L = NA	R = NA; L = NA	1.57	R = 1; L = 1	R = NA; L = NA	R = NA; L = NA
2	2.43	R = 2; L = 3	R = 13; L = 20	R = B; L = B	4.00	R = 2; L = 2	R = 17; L = 35	R = NA; L = NA
3	2.14	R = 3; L = 2	R = 33; L = 25	R = B; L = A	1.00	R = 1; L = 1	R = 20; L = 37	R = A; L = A
4	3.86	R = 3; L = 3	R = 2; L = 5	R = A; L = A	2.14	R = 1; L = 1	R = 2; L = 5	R = A; L = A
5	1.86	R = 3	R = 7	R = NA	2.43	R = 1	R = 5	R = A
6	2.14	R = 3	R = 0	R = A	2.57	R = 2	R = 2	R = A
7	2.43	R = 3; L = 2	R = 7; L = 7	R = A; L = C	1.86	R = 1; L = 1	R = 3; L = 5	R = A; L = A
8	1.29	R = 1; L = 1	R = 17; L = 17	R = A; L = A	1.29	R = 1; L = 1	R = 5; L = 18	R = A; L = A
9	2.86	L = 3	L = 5	L = A	2.14	L = 1	L = −2	L = A
10	4.86	R = 4; L = 4	R = 15; L = 22	R = C; L = C	1.86	R = 1; L = 1	R = 12; L = 8	R = A; L = A
11	2.57	R = 4; L = 4	R = 18; L = 17	R = B; L = B	2.57	R = 4; L = 4	R = 18; L = 13	R = B; L = B
12	2.86	R = 2; L = 3	R = 18; L = 27	R = B; L = B	1.86	R = 3; L = 3	R = 18; L = 32	R = B; L = B
13	4.00	R = 3; L = 4	R = 2; L = 2	R = A; L = A	2.86	R = 1; L = 1	R = 3; L = 5	R = A; L = A
14	4.00	R = 3; L = 3	R = 13; L = 8	R = A; L = A	3.14	R = 1; L = 1	R = 8; L = 8	R = A; L = A
15	1.43	R = 2; L = 2	R = 7; L = 28	R = A; L = B	1.14	R = 1; L = 1	R = 13; L = 28	R = NA; L = NA
16	4.43	R = 4; L = 4	R = 18; L = 33	R = NA; L = B	2.00	R = 2; L = 4	R = 20; L = 33	R = NA; L = B
17	2.71	L = 3	L = 10	L = A	2.43	L = 1	L = 13	L = A
18	4.43	L = 3	L = 27	L = C	3.00	L = 2	L = 30	L = C
19	3.57	R = 3; L = 3	R = 10; L = 8	R = A; L = A	4.71	R = 1; L = 1	R = NA; L = NA	R = NA; L = NA
20	1.43	R = 3; L = 3	R = 12; L = 12	R = A; L = A	1.14	R = 1; L = 1	R = 5; L = 2	R = A; L = A
21	3.00	R = 3; L = 3	R = 18; L = 28	R = C; L = C	2.29	R = 1; L = 2	R = 23; L = 30	R = C; L = C
22	2.43	L = 4	L = 5	L = A	1.71	L = 1	L = 5	L = A
23	2.29	R = 4	R = 70	R = NA	2.86	R = 4	R = 68	R = B
24	3.86	L = 2	L = NA	L = NA	1.71	L = 2	L = NA	L = NA
25	3.57	L = 2	L = 10	L = NA	1.29	L = 1	L = 8	L = B
26	2.29	R = 2	R = 2	R = A	1.71	R = 1	R = NA	R = NA
27	4.57	R = 3; L = 3	R = NA; L = NA	R = A; L = A	3.14	R = 1; L = 1	R = 2; L = 3	R = A; L = A
28	1.86	L = 3	L = 37	L = C	1.29	L = 1	L = 42	L = C
29	1.57	L = 3	L = 23	L = C	2.57	L = 1	L = 15	L = A
30	2.71	L = 2	L = 8	L = A	2.57	L = 1	L = NA	L = A

R, right; L, left; PTA, pure tone average, in dB; ACT, air conduction; Tymp, tympanometry; NA, not available.

Audiograms were available preoperatively for 42 patients and postoperatively for 41 patients. However, bone conduction thresholds were missing for five patients preoperatively and for one postoperatively, following which normal air conduction thresholds were used. A comparison of preoperative and postoperative audiograms revealed no statistically significant difference regarding the pure tone average for air conduction (*P* = 0.56), bone conduction (*P* = 0.95), as well as air bone gap (*P *> 0.79).

There were 40 tympanograms available preoperatively and 36 tympanograms postoperatively. A comparison of these tympanograms, with the exclusion of patients with a tympanic membrane perforation, revealed no statistically significant difference regarding BDET (*P* = 0.07).

Preoperative otoscopic examinations showed an intact tympanic membrane in 45 patients, with one case of t-tube and one case of tympanic membrane perforation. Postoperative otoscopic examinations revealed no new tympanic membrane perforations.

## Discussion

This study demonstrates the feasibility and safety of balloon dilatation of the Eustachian tube as an in-office procedure performed during the COVID-19 pandemic. A comparison of ETMIS and mean overall ETDQ-7 scores 3 months after the intervention showed significant improvement compared with the preoperative scores. However, pre- and postoperative audiometric data showed no significant change.

Balloon dilatation of the Eustachian tube has demonstrated increasing evidence for the effective treatment of tubal ventilation disorders (7). The first endoscopic transnasal surgical approaches included microdebrider (12) and laser Eustachian tuboplasty (13). The first studies of balloon catheter sinuplasty gave birth to the idea of BDET, and initial cadaver studies confirmed its safety and feasibility (14, 15). The most common pathology seems to be functional obstruction due to an inflammation and edema of the mucosa in the fibrocartilaginous part in the valve region of the Eustachian tube (16, 17). Therefore, the effect of balloon dilation consists of a crushing mechanism of the submucosal layer and thinning of the mucosa. A reduction of the submucosal inflammation cells and their replacement with a thin fibrous scar, which result in a stiffening of the tube, are shown. Through these changes, a better dilation and ventilation of the Eustachian tube is reached (18, 19). The first published BDET was performed in 2010 (8). A recent systematic review and meta-analysis demonstrated significantly improved subjective and objective outcomes (7).

The clinical diagnosis of ETD is based on subjective symptoms: otoscopic finding of tympanic membrane retraction and/or negative middle ear pressure on the tympanogram. The ETDQ-7 score has been validated to assess ETD with a normal score at <2.1 (7). The reported normalization of ETDQ-7 scores ranges between 53% and 58% (7). While the majority of patients of our cohort showed a beneficial effect of BDET, two (6.6%) patients showed an unchanged score and seven (23.3%) an increase in the ETDQ-7 score. The ETDQ-7 score is limited by its subjective nature and can be affected by acute respiratory or sinunasal disease. Furthermore, some patients with normal objective findings suffer from baro-challenge-induced ETD, for which there is increased evidence on the efficacy of BDET (20). To date, no predictors of the outcome of BDET are available, and therefore, it remains unclear how many and what type of patients derived greater benefit from this intervention.

Apart from BDET, no evidence is available for knowing the therapeutic effects of topical or systemic medical treatment of ETD (21). Topical nasal steroids are still recommended as initial standard treatment, although there is no evidence of a therapeutic effect to date. Only one randomized, placebo-controlled trial examining the effect of triamcinolone or placebo found no significant difference in the scores related to tympanometry signs or symptoms after 6 weeks (6). Two other studies examining the effects of topical sympathomimetics and systemic sympathomimetics with antihistamines or placebo reported a statistically significant improvement in Eustachian tube function, but only in a small number of participants and in a short period of time, with these limitations aided by the fact that these studies did not assess symptomatic improvement (22, 23). A recent systematic review and meta-analysis confirmed the continuing lack of evidence on the efficacy of medical management options in ETD (24). Therefore, it can be concluded that a further systematic evaluation of medical treatment of ETD in larger, prospective, and randomized-controlled trials is necessary.

Previous studies have reported on the performance of BDET in local anesthesia with different balloon dilation devices or anesthesia. A recent outcome comparison of balloon dilatation under local vs. general anesthesia revealed comparable results for the procedure, regardless of the anesthetic protocol (25). Compared with the modus operandi of previously published BDET under local anesthesia (9, 10, 25), we did not routinely administer local anesthesia in the ET lumen area. All patients of our cohort received local anesthesia with local lidocaine spray and nasal packing, with this being sufficient in 90% of the patients. However, 10% of patients required infiltration anesthesia of the nasal septum and Eustachian tube. BDET as an in-office procedure is already known to be well tolerated in local anesthesia with a previously maximum reported VAS of 6.1 ± 1.0 and 96% willingness to choose local anesthesia again (26). Similar tolerability for a balloon dilatation procedure was reported for balloon sinuplasty under local anesthesia with a score of 4 on the Wong–Baker FACES pain scale (27). Our cohort reported a mean discomfort of 4.7 on the VAS during the intervention, and the recommendation rate for the procedure to be performed, under local anesthesia, on a friend, was 100%. Therefore, performing the intervention in local anesthesia proves to be a valuable alternative in most patients.

We reported one complication of cervico-facial emphysema, which was spontaneously resolved. Other patient cases, with one case of pneumomediastinum, have been reported in the literature. They were all managed conservatively under prophylactic antibiotics (28–30). In the initial days following the intervention, the performance of the Valsalva maneuver must be monitored, and sneezing with closed mouth or lifting heavy weights must be avoided. Because of the proximity of the internal carotid artery to the Eustachian tube, controversy persists as to whether prior computed tomography (CT) is necessary (31). In our cohort, temporal bone CT scans were not performed preoperatively, and a device with a built-in stop mechanism was used. The overall reported complications were rated at approximately 5%, consisting mostly of self-limiting epistaxis. In addition, the procedure has been shown to be safe for the pediatric and postoperative head and neck radiotherapy population and is especially performed in recalcitrant patients (32, 33).

COVID-19 caused significant changes in otorhinolaryngologic daily practice. Functional surgical procedures including BDET were frequently postponed because of limited surgical and nursing resources, with only emergency and oncologic surgeries being performed. Moreover, procedures under general anesthesia were postponed for an indefinite period in the initial stages of the pandemic, resulting in long waiting lists of patients. This was driven in part by the potential risk of infection to healthcare professionals and in part by the use of reduced healthcare resources to avoid potential shortage in such resources. Due to the fact that BDET was performed under general anesthesia before the COVID-19 pandemic, this intervention became a primary candidate for postponement. COVID-19 is mainly a respiratory infection transmitted through droplets and aerosols with the primary infection of differentiated multiciliary cells of the nasal respiratory epithelium (34). For standard rhinological procedures including nasal endoscopy, no droplet generation was found in contrast to the use of powered instruments outside the nasal cavity (35). Similarly, the risk of droplet formation during otologic surgery was assessed (35). During BDET, nasal endoscopy is performed with the subsequent placement of the balloon device under direct visualization in the tubal orifice. Therefore, it might be assumed that balloon dilation of the Eustachian tube also generates no droplet formation. However, droplet formation might result with the use of alternate methods such as microdebrider or laser Eustachian tuboplasty, although this might be minimized with appropriate suction. The use of N95 masks, eye-protection devices, fluid-resistant gowns, and surgical gloves as personal protective equipment is recommended and these were used during the intervention (36). No COVID-19 infections related to BDET were observed in our team.

Performing the procedure under local anesthesia during the COVID-19 pandemic resulted in saved nursing and healthcare resources, as patients were able to return home immediately after the procedure. Furthermore, the anesthesiologic risks of performing the procedure under general anesthesia were eliminated. Nevertheless, this study has potential limitations, as only a relatively small number of patients is included and the reported scores rely on subjective analyses as well as subjective patient questionnaires. Furthermore, no statistically significant difference between preoperative and postoperative tympanograms after BDET were observed. However, this seems to be one of the first uses of BDET in local anesthesia in Europe reporting favorable outcomes during the COVID-19 pandemic.

## Conclusion

In the light of the COVID-19 pandemic, this study illustrates that BDET can be performed in local anesthesia and is well tolerated by most patients. Appropriate patient selection and adequate preoperative information on the procedure are important. The assessment of subjective and objective symptoms with the ETDQ-7 score and ETMIS showed improved outcomes over a follow-up of 3 months. Apart from one complication of self-limiting subcutaneous emphysema, no major complications occurred.

## Data Availability

The raw data supporting the conclusions of this article will be made available by the authors without undue reservation.
